# Efficient Phytase Secretion and Phytate Degradation by Recombinant *Bifidobacterium longum* JCM 1217

**DOI:** 10.3389/fmicb.2019.00796

**Published:** 2019-04-16

**Authors:** Zhongke Sun, Zonghao Yue, Xingdong Yang, Xinqi Hao, Maoping Song, Lili Li, Can Chen, Cuiwei Chu, Chengwei Li

**Affiliations:** ^1^College of Life Sciences and Agronomy, Zhoukou Normal University, Zhoukou, China; ^2^College of Chemistry and Molecular Engineering, Zhengzhou University, Zhengzhou, China; ^3^Key Laboratory of Plant Molecular Breeding and Bioreactor, Zhoukou, China

**Keywords:** *Bifidobacterium longum* JCM 1217, promoter, phytase, phytate, phosphorus

## Abstract

Genetic engineering of probiotics, like bifidobacteria, may improve their microbial cell factory economy. This work designed a novel shuttle plasmid pBPES, which bears exogenous *appA* and is stable within *Bifidobacterium longum* JCM 1217. Cloning of three predicted promoters into pBPES proved that all of them drive appA expression in *B. longum* JCM 1217. Transformation of plasmids pBPES-tu and pBPES-groEL into *B. longum* JCM1217 resulted in much more phytase secretion suggests P*_tu_* and P*_groEL_* are strong promoters. Further *in vitro* and *in vivo* experiments suggested *B. longum* JCM 1217/pBPES-tu degrades phytate efficiently. In conclusion, the study screened two stronger promoters and constructed a recombinant live probiotic strain for effectively phytase secretion and phytate degradation in gut. The strategy used in the study provided a novel technique for improving the bioaccessibility of phytate and decreasing phosphorus excretion.

## Introduction

Bifidobacteria are ideal hosts for food-grade delivery of useful enzymes. Strong promoters can regulate high-level gene expression thereby improving the efficiency of microbial cell factories. However, most of those finely defined promoters that had been used in other hosts cannot be readily applied in bifidobacteria (Sun et al., 2012). Therefore, researchers turn to isolate promoters from bifidobacteria themselves (Sun et al., 2014b). The housekeeping gene glyceraldehyde-3-phosphate dehydrogenase (GAPDH) is widely expressed in both prokaryotic and eukaryotic cells. Therefore, its promoter P*_gap_* was cloned and showed strong capacity for driving high-level expression of several proteins (Klijn et al., 2006; Khokhlova et al., 2010; Osswald et al., 2015a,b). Our previous study revealed that P*_gap_* works in three species of *Bifidobacterium* (Sun, 2014). However, proteomic studies suggest that proteins of elongation factor Tu and groEL are more abundantly expressed than GAPDH in many bacteria, including *B. longum* NCC2705 and *B. bifidum* S17 (Liu et al., 2011; Wei et al., 2016). Hence, their promoters may be stronger than P*_gap_*. *B. longum* JCM 1217 (also known as ATCC 15707) is a typical probiotic strain that protects host from enteropathogenic infection (*Escherichia coli* O157:H7) through production of acetate (Fukuda et al., 2011). The strain also reduced biofilm formation in pathogenic *E. coli*, reduced colitis in rats, and increased survival rate of *Clostridium difficile* infection in mice (Kim et al., 2012; Celiberto et al., 2017; Yun et al., 2017). If this strain was used as a host for foreign gene expression, its economy may improve as their synergistic effects.

Phytate is the main form of phosphorus storage in plant derived foods. However, due to the lack of endogenous phytase enzymes, monogastric animals cannot metabolize phytate (Lei et al., 2013). Therefore, inorganic phosphate is supplemented in diets of these animals to meet their nutritional requirement, while undigested phytate in excreta causes serious pollution. Meanwhile, undigested phytate significantly inhibits the absorption of zinc and iron from grains in both human and animals, leading to mineral deficiencies through the formation of insoluble chelates (Frontela et al., 2008). Further, tumor cells can employ extracellular phytate to improve their proliferation (Windhorst et al., 2013). Currently, there are two techniques for liberating phytate in grains. One is hydrolyzing it through food physicochemical processes, and the other is enzymatic degradation using phytases. Due to activation of the endogenous phytase that presented in plant foods or addition of exogenous phytase, phytate was degraded to various inositolphosphates, containing 1-5 phosphate groups per molecule of inositol. Commercial phytases are mainly produced by *E. coli* and *Aspergillus niger* (*A. niger*). Though these phytases were widely applied for reducing phytate in food and feed, they need purification, which significantly increases cost and causes activity loss during industrial processes (Lei et al., 2013). A few strains of wide-type phytase positive bifidobacteria had been applied in both feed and food for liberating phytate (García-Mantrana et al., 2014, 2015). However, the enzyme activity in these strains is much lower if detectable. Considering the important role of phytase and the increasing interest in its application, as well as available genetic tools in bifidobacteria, it is possible to engineer bifidobacteria for phytase over-expression (Parvaneh et al., 2014; Chen et al., 2016).

To overcome the shortage of genetic tools in *B. longum* JCM 1217 and support our hypothesis, we composed a vector and cloned three promoters for driving heterologous phytase secretion. One recombinant strain *B. longum* JCM 1217/pBPES-tu was tested for effective degradation of phytate *in vitro* and *in vivo*. The study aims at providing a recombinant probiotic strain that can effectively improve phytate accessibility in food or feed industry.

## Materials and Methods

### Bacterial Strains, Plasmids, and Growth Conditions

Type strain of *E. coli* DH5α obtained from Invitrogen was used for general cloning. The strain has standard genotypes and was grown in Luria Bertani (LB) broth at 37°C under agitation. Dry powder of strain *B. longum* JCM 1217 was purchased from Guangdong Institute of Microbiology Culture Center (GIMCC, China). It was propagated routinely in Lactobacilli MRS medium (BD Difco^TM^, United States) supplemented with 0.5 g/L L-cysteine (Sigma-Aldrich, China) or adapted Reinforced Clostridium Medium (RCM, Qingdao Hopebiol Co., Ltd., China) without addition of 0.5 g/L agar (Solarbio Co., Ltd., China). Growth of bifidobacteria was carried out statically at 37°C in sealed jars under anaerobic condition using the MGC AnaeroPack (Mitsubishi^TM^, Japan). Antibiotics and other chemical regents were purchased from Sigma-Aldrich. Chloramphenicol was added at a final concentration of 25 μg/mL for selection in both *E. coli* and *B. longum*. All strains and plasmids used in this study were listed in Table 1.

**Table 1:** Bacterial strains and plasmids used in this study.

Strain or plasmid	Relevant features or description	Sources
*E. coli* DH5a	Type strain	Invitrogen
*B. longum* JCM 1217	Sequenced wide-type strain	GIMCC
*B. longum* JCM 1217/*pBPES*	JCM 1217 possessing plasmid *pBPES*	This study
*B. longum* JCM 1217/*pBPES-tu*	JCM 1217 possessing plasmid *pBPES-tu*	This study
*B. longum* JCM 1217/*pBPES-groEL*	JCM 1217 possessing plasmid *pBPES-groEL*	This study
*B. longum* JCM 1217/*pBPES-gap*	JCM 1217 possessing plasmid *pBPES-gap*	This study
*pBPES*	*Escherichia coli-Bifidobacterium* shuttle vector, no promoter, bearing signal peptide and *appA* gene	This study
*pBPES-tu*	*pBPES* containing 172 bp promoter region of gene elongation factor Tu (*BLLJ_0515*)	This study
*pBPES-groEL*	*pBPES* containing 241 bp promoter region of gene *groEL (BLLJ_1448)*	This study
*pBPES-gap*	*pBPES* containing 240 bp promoter region of gene *GAPDH (BLLJ_1241)*	This study

*GIMCC; Guangdong Institute of Microbiology Culture Center, Guangdong Province, China*.

### Construction of Shuttle Plasmid Backbone

**Figure 1:**
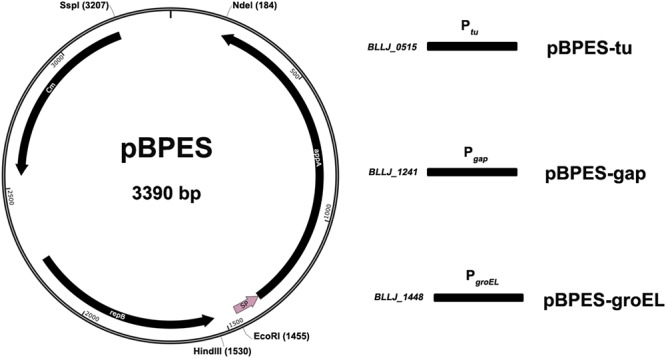
Schematic presentation of plasmid pBPES and its derivatives. Plasmid pBPES was the backbone used for screening promoters. The vector was an *Escherichia coli* – *Bifidobacterium longum* shuttle plasmid based on pUC57 using pMB1 cloned from pTB60 as a replicon, Cm^r^ as a selection marker, SP as a signal peptide. Chloramphenicol was used for selection of the plasmid at a concentration of 25 μg/mL in both *E. coli* and *B. longum.* P*_tu_*, P*_gap_*, and P*_groEL_* were predicted promoters of gene *BLLJ_0515*, *BLLJ_1241*, and *BLLJ_1448*, respectively. They were cloned into pBPES between restriction sites of Hind III and EcoR I. The resulted three derivative plasmids were named as pBPES-tu, pBPES-gap, and pBPES-groEL, respectively.

The plasmid backbone of pBPES was constructed as presented (Figure 1). The chloramphenicol resistance gene Cm^r^ is a selection marker in both *E. coli* and *Bifidobacterium*. A pMB1 replicon derived from the bifidobacterial plasmid pTB60 preserves its replication in *B. longum* JCM 1217. BBIF_1761 was a surface protein and its signal peptide (SP) directed efficient secretion of appA in both *B. longum* E18 and *B. bifidum* S17 (Osswald et al., 2015b). Fukuda et al. reported that a cell surface protein (BLLJ_1900) in the genome of *B. longum* JCM 1271 has the highest similarity to protein BBIF_1761 (Fukuda et al., 2011), shown by protein Blast analysis. Therefore, the predicted SP encoding sequence of gene *BLJJ_1900* was cloned (see SP prediction and detail sequence in Supplementary Information). Briefly, the SP sequence of BLLJ_1900 was amplified by PCR using primers SPF/SPR with JCM 1217 genome as template. The parameters for PCR were pre-denaturated at 98°C for 3 min; then 35 cycles by denaturing at 95°C for 10 s, annealing at 55°C for 10 s and elongation at 72°C for 30 s. The mature form of phytase encoding gene *appA* was amplified by PCR using primers PhyF/PhyR with *E. coli* DH5α genome as a template according to a previous report (Sun, 2014). The 1009 bp replicon *repB*, including its promoter region of plasmid pTB6, was chemically synthesized according to its sequence as the shortage of DNA template (Tanaka et al., 2005). The 660 bp antibiotic resistance gene catA1 (Cm^r^) was chemically synthesized as well (Accession No.: NG_047582.1). All chemical DNA synthesis service was provided by a commercial supplier (Nanjing GenScript Co., Ltd., China). After all DNA elements were obtained, SOEing PCR was conducted using primers Soe1F/Soe1R with the mixture of *SP* and *appA* or using primers Soe2F/Soe2R with the mixture of *Cm^r^* and *repB* fragments in equal molar as templates. The SOEing PCR experiments were performed for five cycles without primers and continued 25 cycles with primers. Parameters were similar to traditional PCR except the elongation time extended to 2 min. The commercial plasmid pUC57 and the amplicon *Cm^r^-repB* obtained through SOEing PCR were digested by Ssp I and Hind III and then ligated to form an intermediate cloning plasmid pBS. The plasmid pBS and the amplicon *SP-appA* were further digested by EcoR I and Nde I. Digested plasmid and amplicon were ligated for framing Bifidobacterial Phytase Expressing Shuttle vector pBPES, which will be used as a backbone for promoter screen. All molecular enzymes, including that used for restriction digest and ligation were purchased from NEB (New England Biolabs Ltd., Beijing, China).

### Prediction and Cloning of Promoters

Potential promoter regions of genes *BLLJ_0515* (Elongation factor Tu), *BLLJ_1241* (GAPDH), and *BLLJ_1448* (groEL) were predicted by BPROM^1^ with their upstream intergenic sequences. Full intergenic sequences, including predicted core promoter regions spanning from 172 to 241 bp, were amplified with specific primers using *B. longum* JCM 1217 genomic DNA as a template (e g., primers tuF/tuR for P*_tu_*, primers gapF/gapR for P*_gap_*, and primers groF/groR for P*_groEL_*). Then, they were digested and inserted into plasmid pBPES between restriction sites Hind III and EcoR I. Genomic DNA of *B. longum* JCM 1217 was extracted by a Rapid Bacterial Genomic DNA Isolation Kit (Sangon Biotech Co., Ltd., China). All DNA fragments were purified by the Universal DNA Purification Kit (Beijing Tiangen Biotech Co., Ltd., China). Transformation of *E. coli* DH5α and *B. longum* JCM 1217 was achieved through electroporation as described (Sun, 2014). Recombinant constructs were screened by colony PCR with primer PMF/PMR. Plasmid DNA was extracted with Plasmid Mini Extraction Kit (Tiangen Biotech. Co., Ltd., China) and sequenced by ABI3730 (Nanjing Genscript Biotech Co., Ltd., China). Sequence alignment was conducted with local BLAST program to make sure there are no mutations in promoter regions and open reading frames are correct. All primers used in this study were designed with Primer3 online and synthesized by a commercial supplier (Nanjing Genscript Biotech Co., Ltd., China) as listed (Table 2).

**Table 2:** Primers used in this study.

Primer	Nucleotide sequence	Amplicon	Size (bp)
SPR	ATCCGCTGCGTTGGCCGTG	SP	99
SPF	ATGAAATCACTGATGAAAAAGG		
PhyR	TTACAAACTGCACGCCGGT	mature appA	1233
PhyF	CAGAGTGAGCCGGAGCTGA		
Soe1R	GGAATTCCATATGTTACAAACTG CACGCCGGT	SP-appA	1353
Soe1F	CGGAATTCATGAAATCACTGAT GAAAAAGG		
Soe2R	CCCAAGCTTTCAAACGCGCGA GAAACGGCATT	Cm^r^ -pMB1	1669
Soe2F	CGAATATTATGGAGAAAAAAATC ACTGGATATAC		
tuR	CGGAATTCTACTTTTGTCCTCCT GGACGTCTC	P*_tu_*	172
tuF	CCCAAGCTTCACGCGCCACTG CATGAAG		
gapR	CGGAATTCGTAATTCTCCCTTGT AGGGTGG	P*_gap_*	240
gapF	CCCAAGCTTCTTGCATGCCGC GCGCTTG		
groR	CGGAATTCGAGTAACACGCAC GCAAGGATG	P*_groEL_*	241
groF	CCCAAGCTTATGGTTGCGTATT CCTCCAAATGGCT		
rPR	ATCCGCTGCGTTGGCCGTG	promoter-	variable
		SP-appA
rPF	TCCGGAGACGTCAGCTGCT		

*Underlined: digestion sites for restriction enzymes*.

### Enzymatic Assay of Recombinant Phytase Secretion in *B. longum* JCM 1217

All samples used for enzyme assays were prepared as described using ultra-pure water (Osswald et al., 2015b). Briefly, cell free medium (CFM) was prepared by collection of supernatants after centrifugation (5000 g, 5 min). Cell pellets were rinsed twice in 0.2 M sodium citrate buffer, pH5.5, and then disrupted using 0.1 mm glass beads (Sigma-Aldrich, China) by homogenization (Scientz-48, Ningbo, China). Then, total protein concentration in the cell extract was determined by the Pierce BCA Protein Assay Kit (Thermo Fisher Scientific, Germany). Phytase assay was adapted from the Phytex method described elsewhere with minor changes (Kim and Lei, 2005). In brief, 100 μL pre-warmed supernatant of cell crude extract was mixed with equal volume 10.8 mM sodium phytate. Reaction lasted for 15 min at 37°C, and then stopped by 200 μL 15% tricholoroacetic acid. After centrifugation at 12000 rpm for 2 min, 20 μL supernatant was removed into 980 μL color solution (fresh mixture of 1 M sulfuric acid, 2.5% (w/v) ammonium molybdate in ddH_2_O, and 10% (w/v) ascorbic acid in ddH_2_O at a ratio of 3:1:1). Absorbance at 820 nm was read by a spectrophotometer (UV722N, Shanghai Yoke Instrument Ltd., China) in 1 cm plastic cuvettes. Phytase activity was calculated according to a standard curve using 9 mM potassium dihydrogen phosphate in water. One phytase unit (FTU) was defined as an enzyme that catalyzes the release of 1 μmol inorganic phosphate per minute in the above condition. Specific activities were expressed as FTU/mg in cell lysate and FTU/mL in CFM.

### Phytate Degradation by *B. longum* JCM 1217/pBPES-tu *in vitro*

Phytate degradation *in vitro* was first confirmed as described by spotting bacteria on the agar plate of phytase screen medium, which supplemented insoluble Ca-phytate as a substrate (Sun, 2014). After 48 h incubation at 37°C, clean zone around colony was an obvious sign of phytate degradation. Second, the CFM was collected after growth of the recombinant strain *B. longum* JCM 1217/pBPES-tu in modified RCM broth, and was tested for degradation of 0.2% (w/v) maize powder (yellow, cultivar Huanong 101, provided by Da Bei Nong Group, Zhoukou, China), as P*_tu_* is the strongest promoter in this study (Sanz-Penella et al., 2009). After fermentation of *B. longum* JCM 1217/pBPES-tu statically in the adapted RCM broth at 37°C for 48 h, spent CFM was prepared by centrifugation (10000RCF for 10 min) and stored at 4°C before assay. Degradation of phytate in maize powder was measured by test of released inorganic phosphate in CFM. Uncultured medium was used as a negative control, and commercial enzyme (Szym-PHY10G, SinoBios Imp. & Exp. Co., Ltd., Shanghai) was used as a positive control (PC). Third, *B. longum* JCM 1217/pBPES and *B. longum* JCM 1217/pBPES-tu were inoculated into adapted RCM supplemented 0.2% maize powder. Final bacterial counts and inorganic phosphorus in CFMs were measured after 48 h incubation. Bacterial counts were determined by plating of 100 μL culture fluids on the surface of MRS agar after 48 h incubation anaerobically. Soluble inorganic phosphorus was assayed as above enzymatic assay and calculated according to the same standard curve.

### Phytate Degradation by *B. longum* JCM 1217/pBPES-tu *in vivo*

Animal experiments were approved by the academic committee of Zhoukou Normal University. Forty-five 1-day-old Arbor Acres (AA) male broilers were randomly allocated into nine cages, with five in each cage, and were managed according to the Arbor Acres Management Guide (Manual 2009^2^). All birds were fed a mixture of 1.9 kg commercial premix meal (LNB^TM^, Cargill, China) and 0.1 kg maize powder per kg of chicks’ body weight gain each day, unless indicated otherwise. The commercial premix meal is based on corn-soybean (55.3%: 38%) and contains 3% fish oil, 1.4% calcium hydrophosphate, 1% limestone, 0.3% salt, and 1% additives in extra (all in w/w). Cages 1–3 were assigned to non-treat group (NG), in which animals were always fed above the indicated meal. Cages 4–6 were assigned to test group (TG), in which animals were fed an adjusted diet that contains 10^9^cfu *B. longum* JCM 1217/pBPES-tu per kg of meal for 2 weeks and then readapted to the diet used in NG group for two further weeks. Cages 7–9 were assigned to the control group (CG), who were fed an adjusted diet containing 10^9^cfu *B. longum* JCM 1217/pBPES per kg of meal for 2 weeks and then readapted to the diet used in NG group for a further 2 weeks. Adjusted diets containing bacteria were prepared as previously described, using a granulating machine (GL-25A, Ronghua Machinery Manufactory Co., Ltd., China) (Li and Shi, 2016). Briefly, to produce enough bacterial cells, different recombinant *B. longum* were fermented in 1L MRS broth supplemented with antibiotics for 48 h, respectively. Bacterial cell pellets were collected by centrifugation and then double-washed by ddH_2_O. Then, bacterial cells suspended in 100 mL water were mixed with premix meal and maize powder at a certain ratio (2 × 10^9^CFU bacteria: 1.9 kg premix meal: 0.1 kg maize powder). At last, adjusted diets were processed by a granulating machine before feeding. The weights of adjusted diets were 5% higher than that of un-supplemented diet due to the supplementation of bacterial suspension. Supplementation of bacteria has a negligible influence on the concentrations of total phosphorus in all diets and did not affect the body weight gain of birds after feeding (as shown in Supplementary Table S2). Plentiful distilled water was always supplied, and birds were raised under controlled conditions similar to that in commercial practiced during the study in a farm (Shandong Good Earth Company, China).

### Assay of Total Phosphorus and Remnant Phytate Contents in Feces

During the experiment, live weights of all chicks in each cage were recorded daily and the body weight gain was calculated. Feces of chicks in each cage were collected at each weekend and dried in an oven at 60°C for 24 h before assay. Contents of total phosphorus (TP) in fecal samples were assayed according to a previous report (Bremer et al., 2008). Briefly, 1 g feces were ashed for 6 h and extracted with 10 mL of 3 N HCl. Then, water diluted extracts were filtered through filter paper (Whatman 42; 2.5 μm) for colorimetric analysis in duplicate with the molybdovanadate method by reading absorbance on a spectrophotometer (MultiScan FC, Thermo Fisher Scientific) and calibrated with standards developed in a similar procedure. Remnant phytate in feces was extracted and quantified by high performance liquid chromatography, according to a previous report with minor modification during extraction (Hotz and Gibson, 2001). Briefly, feces were dried in a 90°C oven until a constant weight was reached. Then, 5 g dried sample was extracted with 5 mL of 0.67 M HCl by vortexing in a metal bath at 37°C for 20 min. Following centrifugation at 5000RCF for 10 min, 2.5 mL of the supernatant was removed and mixed with 2.5 mL of ddH_2_O water. For parallel comparison, the relative contents of TP and remnant phytate were normalized by their contents in the feces of NG and expressed as percentages.

### Isolation and Verification of Recombinant Bifidobacteria in Feces

To confirm the viability and possible colonization of engineered bacteria *in vivo*, strains of recombinant bifidobacteria were isolated from feces by culture-based method. Briefly, freshly collected feces at each weekend were suspended in sterile saline and then vortexed for 2 min. Supernatants obtained after centrifugation at 3000RCF for 2 min were spread on MRS agar supplemented with 25 μg/mL chloramphenicol. After anaerobical incubation at 37°C for 48 h, ten visible colonies were randomly picked and were amplified by colony PCR using primers rPF/rPR, which are specific sequences derived from plasmid pBPES (primers see Table 2). PCR positive colonies were further verified by enzymatic assays using a F6PPK ELISA Kit according to the user guide (Qingdao Hopebiol Co., Ltd., China).

### Data Analysis

For all *in vitro* data, the mean of three independent experiments with triple replicates were calculated and expressed as mean ± standard deviations (SD). All *in vivo* data were obtained from three cages and expressed as relative mean ± SD. Statistical analysis for *in vitro* degradation of phytate was performed using Student’s *t*-test (significant with *P* < 0.01). Statistical analysis for *in vivo* study was performed using SPSS19.0 by one-way ANOVA with Bonferroni post-tests.

## Results and Discussion

### Stability of Shuttle Plasmid pBPES and Cloning of Predicted Promoter

The plasmid pBPES was constructed as designed in this study. Transformation into and successful isolation from both *E. coli* DH5α and *B. longum* JCM 1217 suggest that pBPES is indeed a shuttle vector that can be maintained in these hosts. The segregational stability of this plasmid in *B. longum* JCM 1217 was further evaluated by propagation in liquid MRS without antibiotics and the recovery rate under antibiotics reaches 100% after 100 generations and surpasses 95% after 500 generations (data not shown). High recovery rate indicates plasmid pBPES is very stable in JCM 1217 and can be used as a backbone for the construction of expression vectors. BPROM prediction of intergenic sequence yielded one potential promoter for each gene (Supplementary Table S1, Elongation factor Tu, GAPDH, and groEL, see detail results in Supplementary Information). Cloning of these predicted promoters into pBPES resulted in three expressional plasmids, namely pBPES-tu, pBPES-gap, and pBPES-groEL, respectively (Figure 1). Sequencing of the regions spanning promoter and *appA* confirmed that they were in correct open reading frames (ORFs).

### P_tu_ Is a Strong Promoter in *B. longum* JCM 1217

**Figure 2:**
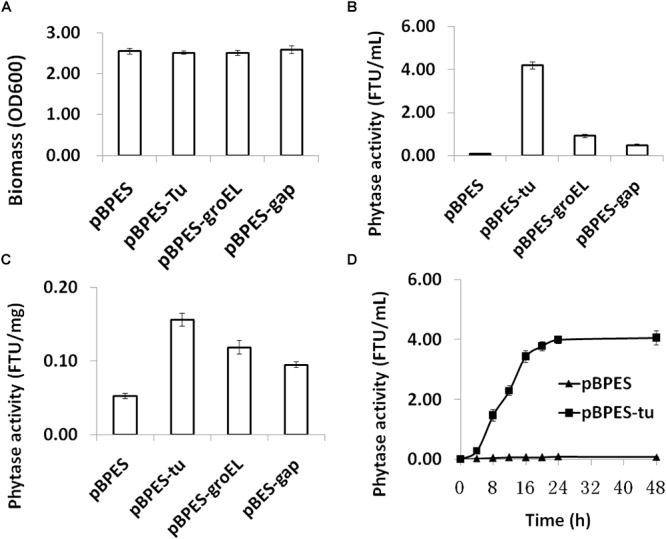
Biomass and phytase expression of recombinant *B. longum* JCM 1217 grown in modified reinforced clostridia medium. **(A)** biomass of different strains assayed by reading optical density at 600 nm after anaerobic incubation for 48 h; **(B)** phytase activity in the CFMs after 48 h incubation of different strains; **(C)** phytase activity in the cell lysate after 48 h incubation of different strains; **(D)** time course phytase activity in CFMs of recombinant *B. longum* JCM 1217 harboring plasmid pBPES or pBPES-tu. Samples were collected at different time points during fermentation in the modified RCM. CFM, cell free medium; RCM, reinforced clostridia medium.

To compare the strength of these three promoters, they were transformed into *B. longum* JCM 1217, respectively. As expected, transformation of these plasmids into *B. longum* JCM 1217 does not influence growth in adapted RCM as all recombinant *B. longum* JCM 1217 strains have a similar biomass after incubation (Figure 2A). The strength of cloned promoters was tested by evaluating *appA* encoded phytase activity in *B. longum* JCM 1217. As shown, phytase activity was detected in the CFMs of all recombinant strains after 48h static incubation (Figure 2B). In fact, all promoters must constitutively drive *appA* expression in *B. longum* JCM 1217 as all constructs have high phytase activity in their CFMs. Among these three promoters, P*_tu_* is significantly stronger than P*_groEL_* and P*_gap_*, though the last is the most extensively used promoter in bifidobacteria (Klijn et al., 2006; Shkoporov et al., 2008; Sun et al., 2014a; Osswald et al., 2015a,b). As shown, activities of phytase in the CFMs of JCM 1217/pBPES-tu and JCM 1217/pBPES-groEL are much higher than that in JCM 1217/pBPES-gap, which reaches 0.48 FTU/mL (Figure 2B). This value is comparable to a previous report in another bifidobacteria strain (Osswald et al., 2015b). Low phytase activity in *B. lognum* JCM/pBPES suggests wide-type bifidobacteria are either negative in phytases or inefficient in degrading phytate, agreeing with a previous report (Palacios et al., 2008). Further time course experiments suggest *B. lognum* JCM 1217/pBPES-tu has the highest level of relative activity at their exponential phases (Figure 2D). Therefore, the study showed promoters of other housekeeping genes, like groEL and elongation factor Tu, are stronger than P_gap_ To be precise, P_tu_ and P_groEL_ are 8- and 1-fold stronger than P_gap_.

### Signal Peptide of BLLJ_1900 Directs Efficient Phytase Secretion in *B. longum* JCM 1217

The secretion of gene products is important for the functionality of many heterogonous expressed enzymes, especially when substrate bioavailability is low, like phytate. It was demonstrated that the SP of cell surface protein BBIF_1761 directs the efficient secretion of appA in *B. bifidum* S17 and *B. longum* E18 (Sun, 2014; Osswald et al., 2015b). Therefore, the predicted SP encoding sequence of its equivalent gene in *B. longum* JCM 1271 was cloned and fused ahead of the mature form of the *appA* gene. Phytase activity is much higher in CFMs and detectable but much lower in cell lysate, suggesting effective secretion of appA (Figure 2B,C). The results also indicated that the SP of BLLJ_1900 directs efficient secretion of appA in *B. longum* JCM 1271.

### *B. longum* JCM 1217/pBPES-tu Degrades Phytate *in vitro*

**Figure 3:**
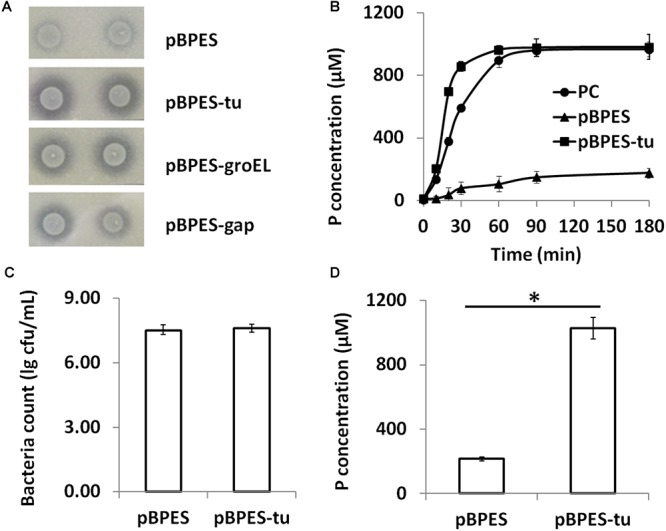
Phytate degradation by *B. longum* JCM 1217/pBPES-tu *in vitro.* **(A)** degradation of phytate-Ca on solid phytase screen medium by spotted bacterial colonies harboring plasmid pBPES or pBPES-tu; **(B)** degradation of phytate from maize powder in liquid phase by CFMs of different recombinant strains or commercial phytase; **(C)** bacteria counts of different recombinant strains grown in the modified RCM plus 2% maize powder; **(D)** degradation of phytate from maize powder by inoculation of different recombinant strains into the modified RCM. All data were mean of three independent experiments with triple replicates and expressed as mean ± SD. Statistical analysis for degradation of phytate was performed using Student’s *t*-test (^∗^significant with *P* < 0.01). CFM, cell free medium; RCM, reinforced clostridia medium; PC, 1 FTU/mL commercial phytase.

Phytate is a main anti-nutritional ingredient in plant-derived diets for all monogastric animals and humans (Schlemmer et al., 2010). Currently, phytase degradation is one of the leading approaches for liberating phytate (Vohra and Satyanarayana, 2003; Jones et al., 2010). Although a few wide-type bifidobacteria strains are phytase positive, their activities are low (Haros et al., 2007, 2009; Oh and Lee, 2007; Sanz-Penella et al., 2012). In the present study, primary phytase degradation by engineered bifidobacteria was displayed phenotypically on the PSM agar containing insoluble Ca-phytate. Clear degradation zones were presented around colonies of *B. longum* JCM 1217/pBPES-tu but were presented much more weakly in *B. longum* JCM 1217/pBPES (Figure 3A). Secondly, the degradation potential of phytase-secreting *B. longum* JCM 1217/pBPES-tu on phytate was evaluated by adding 2% (w/v) maize powder into the spent CFM. The CFM of *B. longum* JCM 1217/pBPES resulted in a much faster degradation of phytate compared to the equal amount of commercial phytase did, though they both finally degraded phytate to similar levels (Figure 3B). Thirdly, strains of recombinant bifidobacteria were inoculated into the adapted medium (RCM) supplemented with 2% (w/v) maize powder. The medium had no influence on the growth as both recombinant strains have similar final bacteria counts (Figure 3C). Meanwhile, inoculation of *B. longum* JCM 1217/pBPES does not lead to obvious phosphate release. In contrast, inoculation of *B. longum* JCM 1217/pBPES-tu resulted in a higher concentration of phosphate in CFM, suggesting effective degradation of phytate in maize (Figure 3D). All these data confirmed that *B. longum* JCM 1217/pBPES-tu can degrade phytate *in vitro*.

### *B. longum* JCM 1217/pBPES-tu Degrades Phytate *in vivo*

**Figure 4:**
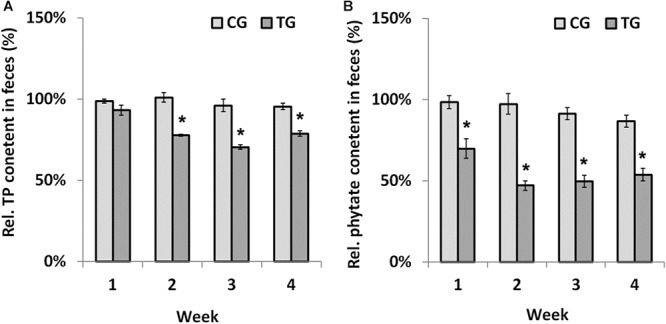
Phytate degradation by *B. longum* JCM 1217/pBPES-tu in chicks. **(A)** relative TP contents in the feces of chicks collected from different groups at each weekend; **(B)** relative remnant phytate in the feces of chicks collected from different groups at each weekend. Data are mean ± SD of three samples collected from three cages relative to the corresponding contents in NG group. Statistical analysis was performed using one-way ANOVA with Bonferroni post-test (^∗^significant with *P* < 0.01). NG, non-treat group; CG, control group; TG, test group.

Chicks were fed recombinant *B. longum* JCM 1217/pBPES-tu to evaluate its effectiveness on phytate degradation *in vivo*. Different diets had little influence on daily body weight gain of chicks during the survey (Supplementary Table S2). The result is in line with a former report that fed broilers with recombinant lactobacilli (Li and Shi, 2016). During treatment, the contents of TP and remnant phytate in feces were assayed at each weekend to reflect the extent of phytate degradation. The contents of TP didn’t differ between NG and CG groups, which suggested that *B. longum* JCM 1217/pBPES has little impact on the excretion of phosphorus (Supplementary Table S3). In contrast, the relative content of TP in the TG group is significantly lower than that in CG group from the second week to the end of the survey (Figure 4A, marked by asterisk, *P* < 0.01). The relative content of remnant phytate is also significantly lower in TG group than that in CG group during the whole treatment (Figure 4B, marked by asterisk, *P* < 0.01). These results indicated that *B. longum* JCM 1217/pBPES-tu degraded phytate effectively *in vivo*. Unexpectedly, at the weekend of the 4th week, the content of remnant phytate in CG group is also significantly lower than that in the NG group (Supplementary Table S4, marked by superscript a, *P* < 0.01). *B. longum* JCM 1217/pBPES-tu degraded phytate *in vivo* is in line with earlier studies using either purified phytases or other microbes producing phytases (Simons et al., 1990; Sanz-Penella et al., 2012; Li and Shi, 2016). However, conflicted results exist as *B. longum* JCM 1217/pBPES was also shown to reduce phytate excretion *in vivo* at the end of the survey, even though no obvious phytase activity can be detected *in vitro*. Although there is a 5% difference in weight between adjusted diets and un-supplemented diet, no difference in both TP and remnant phytate contents in NG and CG groups can be detected during the first 2 weeks of treatment with either diet. But F6PPK positive colonies were grown on MRS supplemented with antibiotics all over the survey even when the CG group was restored to the basal diet. Therefore, detection of recombinant bifidobacteria suggested either that *B. longum* JCM 1217/pBPES slowly colonized the gut or that the pBPES plasmid was horizontally transferred to endogenous bifidobacteria during the 2 week intake. Whatever the pathway, the slow decrease in phytate excretion cannot be related to the sole slow plasmid establishment in the gut as it is deprived in phytase code. It could, however, reflect the promotion of phytase-producing bacteria in the microbiota.

## Conclusion

In conclusion, this study constructed a recombinant strain *B. longum* JCM 1217/pBPES-tu that constitutively secretes phytase appA and confirmed the effectiveness of this strain on phytate degradation both *in vitro* and *in vivo*. The study pioneered the potential of live genetically modified probiotics in the feedstuff industry, and also provided a novel strategy for solving the problems of phosphate utilization and pollution. However, application of this recombinant strain in the food industry is restrained due to legal issues and because further improvement is necessary.

## Ethics Statement

This study was carried out in accordance with the recommendations of Arbor Acres Management Guide, and the protocol was approved by the Animal Ethic and Welfare Committee of Zhoukou Normal University. For this study, involving animals, all institutional and national guidelines for the care and use of laboratory animals were followed.

## Author Contributions

ZS and CL designed the experiments and wrote the manuscript. ZS, ZY, XY, and CaC carried out the experiments. XH, MS, and CuC analyzed experimental results. LL provided technique support and discussed results.

## Conflict of Interest Statement

The authors declare that the research was conducted in the absence of any commercial or financial relationships that could be construed as a potential conflict of interest.

## Footnotes

1 www.softberry.com

2 www.aviagen.com
