# Supplementary material for: Efficient Phytase Secretion and Phytate Degradation by Recombinant Bifidobacterium longum JCM 1217
Source: Front Microbiol. 2019 Apr 16;10:796. doi: 10.3389/fmicb.2019.00796 (PMC6476914; doi:10.3389/fmicb.2019.00796)
Supplement: Supplementary file 1 [file Table_1.docx]

**Supplementary information**

Tables

*Table S1 Potential promoter sequence size and BPROM predicted core features*

| Promoter | P*_tu_* | P*_gap_* | P*_groEL_* |
| --- | --- | --- | --- |
| gene | *BLLJ_0515* | *BLLJ_1241* | *BLLJ_1448* |
| size (bp) | 172 | 240 | 241 |
| -10 region | CAGTAGAAT | CATTACAGT | TGCTAATGT |
| -35 region | GTGGCA | TTGCCA | TTGGCA |
| Known TF binding site | rpoD17: AGTAGAAT | No | No |

TF, transcription factor

*Table S2 The mean body weight gain of broilers in each groups per cage*

| Day | NG | CG | TG |
| --- | --- | --- | --- |
| 2 | 55±2.2 | 56±1.5 | 56±3.1 |
| 3 | 70±1.8 | 71±2.3 | 71±1.6 |
| 4 | 93±2.5 | 95±1.4 | 95±2.7 |
| 5 | 101±4.2 | 102±2.8 | 101±3.5 |
| 6 | 118±3.5 | 120±4.1 | 121±2.6 |
| 7 | 130±2.6 | 131±5.3 | 132±4.1 |
| 8 | 141±3.2 | 142±4.5 | 145±2.4 |
| 9 | 160±4.3 | 163±2.2 | 164±1.8 |
| 10 | 168±5.1 | 169±6.3 | 170±4.2 |
| 11 | 180±3.6 | 183±7.4 | 183±3.3 |
| 12 | 190±6.5 | 192±3.1 | 194±2.4 |
| 13 | 205±8.3 | 208±7.6 | 209±4.9 |
| 14 | 215±4.1 | 218±3.5 | 219±6.2 |
| 15 | 231±7.2 | 232±5.4 | 233±3.8 |
| 16 | 241±3.5 | 243±6.2 | 242±4.7 |
| 17 | 249±5.1 | 252±8.4 | 251±6.3 |
| 18 | 260±2.5 | 261±6.1 | 262±3.4 |
| 19 | 271±7.2 | 273±3.4 | 273±5.6 |
| 20 | 282±4.9 | 282±6.2 | 283±3.3 |
| 21 | 291±8.2 | 292±4.5 | 292±6.1 |
| 22 | 302±5.5 | 305±7.1 | 303±4.3 |
| 23 | 305±3.7 | 308±4.2 | 306±7.5 |
| 24 | 312±6.8 | 315±5.9 | 315±6.2 |
| 25 | 321±7.2 | 323±5.6 | 322±8.9 |
| 26 | 330±10 | 331±8.6 | 332±3.5 |
| 27 | 335±5.6 | 336±9.2 | 337±11 |
| 28 | 340±12 | 342±8.6 | 341±9.4 |

Note: The daily body weight of broilers in each cage was monitored in the morning. Body weight gain (g) was calculated and expressed as mean±SD of five birds from three independent cages. Statistical analysis was performed using SPSS19.0 by one-way ANOVA with Bonferroni post-tests for multiple comparisons. No groups are significantly different from the other (significant with *P* < 0.01). NG, non-treat group; CG, control group; TG, test group

*Table S3 The content of total phosphorus in feces after feeding different diets*

| Treat | Week 1 | Week 2 | Week 3 | Week 4 |
| --- | --- | --- | --- | --- |
| NG | 0.84±0.04 | 0.92±0.06 | 1.03±0.04 | 1.20±0.04 |
| CG | 0.83±0.01 | 0.93±0.03 | 0.99±0.04 | 1.15±0.03 |
| TG | 0.78±0.03 | 0.72±0.01^a, b^ | 0.75±0.02^a, b^ | 0.95±0.02^a, b^ |

Note: Total phosphorus (g/kg) was assayed by colorimetric analysis in duplicate with the molybdovanadate method. Data are mean±SD of samples collected from three cages. Statistical analysis was performed using SPSS19.0 by one-way ANOVA with Bonferroni post-tests for multiple comparisons. Superscript a means significantly different to NG group, and superscript b means significantly different to CG group (significant with *P* < 0.01). NG, non-treat group; CG, control group; TG, test group

*Table S4 The content of remnant phytate in feces after feeding different diets*

| Treat | Week 1 | Week 2 | Week 3 | Week 4 |
| --- | --- | --- | --- | --- |
| NG | 0.64±0.02 | 0.72±0.03 | 0.84±0.03 | 0.93±0.03 |
| CG | 0.63±0.03 | 0.71±0.05 | 0.77±0.03 | 0.81±0.04^a^ |
| TG | 0.45±0.04 ^a, b^ | 0.34±0.02 ^a, b^ | 0.42±0.03 ^a, b^ | 0.50±0.04 ^a, b^ |

Note: Remnant phytate (mg/kg) in feces was quantified by high performance liquid chromatography. Data are mean±SD of samples collected from three cages. Statistical analysis was performed using SPSS19.0 by one-way ANOVA with Bonferroni post-tests for multiple comparisons. Superscript a means significantly different to NG group, and superscript b means significantly different to CG group (significant with *P* < 0.01). NG, non-treat group; CG, control group; TG, test group

Sequences and related annotation

*1. Complete Sequences and predicted details of three potential promoters*

P_tu_

>AP010888.1:666402-666573, BLLJ_0515

CACGCGCCACTGCATGAAGTAGCCAGTGTCTTCAGTCAGCGGTAATATCGTATACCGCTGACTGGGTTCTGGCTCTTCAAAGTGGCACATAACCCAGAAACCCAGTAGAATAAGCGAGTTCCTGTGCGAAAGGGTGCAGGAAACTCACGAGACGTCCAGGAGGACAAAAGTA

BPROM results:

Length of sequence- 172

Number of predicted promoters - 1

Promoter Pos: 118 LDF- 1.84

-10 box at pos. 103 CAGTAGAAT Score 59

-35 box at pos. 82 GTGGCA Score 5

Oligonucleotides from known TF binding sites:

For promoter at 118:

rpoD17: AGTAGAAT at position 104 Score - 11

P_gap_

>AP010888.1:1452498-1452737, BLLJ_1241

CTTGCATGCCGCGCGCTTGCTCATGATGATTCGAGACATTCCTCCAAAGGAGAGGAAAAATCACATGTCGGTTGCGGGCAATCCGAATTGTGAGCGCTCACAGAAAATACGGCATTTTTGCCCAAAACGCACGCTGAAACGTTGCCATGTGTACAGAGTCGGCATTACAGTAGCAACTGTTGGTAAACAATGGCCCGGTGTGCCAAAGCGCGCCAAGGCCACCCTACAAGGGAGAATTAC

BPROM results:

Length of sequence- 240

Threshold for promoters - 0.20

Number of predicted promoters - 1

Promoter Pos: 178 LDF- 1.94

-10 box at pos. 163 CATTACAGT Score 35

-35 box at pos. 142 TTGCCA Score 61

Oligonucleotides from known TF binding sites:

No such sites for promoter at 178

P_groEL_

>AP010888.1:c1721993-1721753, BLLJ_1448

GAGTAACACGCACGCAAGGATGTGCATTGAAGGCCCTGACCGTTTCGACGGTTGGGGCTTTTTCATGCCCAAAGCGCAATCGCCGCCGATGGTGTTGGCACTCGGCGGGCGCGAGTGCTAATGTCTGGGTTAGCACTCGGAGGTCGAGAGTGATAATCGGCAGCTGACGGCTGGTCGTCACGACTCGACGCTTGCGAGTGATAACCGGTATACCTAGCCATTTGGAGGAATACGCAACCAT

BPROM results:

Length of sequence- 241

Threshold for promoters - 0.20

Number of predicted promoters - 1

Promoter Pos: 131 LDF- 0.93

-10 box at pos. 116 TGCTAATGT Score 43

-35 box at pos. 95 TTGGCA Score 38

Oligonucleotides from known TF binding sites:

No such sites for promoter at 131

*2. Signal peptide-encoding sequence used in the study*

ATGAAATCACTGATGAAAAAGGTTTTCGCTGCTGCCGCGGCGATTGCCACCGTATTTGGATTGGCCGCGACGACAGTCGCCACGGCCAACGCA*GCGGAT

* predicted cleavage site by SignalP 6.0
